# International Overdose Awareness Day — August 31, 2018

**DOI:** 10.15585/mmwr.mm6734a1

**Published:** 2018-08-31

August 31, 2018, is International Overdose Awareness Day, a global event to raise awareness that death from drug overdose is preventable. Goals include increasing awareness about the risk for overdose, reducing stigma associated with drug overdose deaths, providing information about community services, and preventing and reducing drug-related harm by supporting evidence-based policy and practice (https://www.overdoseday.com).

The opioid overdose epidemic, which killed over 42,000 Americans in 2016 (*1*), has included three interrelated waves since 1999 (*2*). The first included increases in overdose deaths related to prescription opioids. In 2010, the second wave began and was characterized by a rapid increase in deaths involving heroin. The third and current wave, which began in 2013, is associated with a rapid increase in deaths involving synthetic opioids, such as illicitly manufactured fentanyl and fentanyl analogs. Varying circumstances have been associated with each of these waves, and their intersection presents a unique challenge for a focused and comprehensive response.

Opioid deaths, particularly those involving illicit opioids, continue to increase. As described in a report in this issue of *MMWR*, illicit opioids were detected in approximately three of four opioid overdose deaths compared with nearly four of 10 for prescription opioids in 11 states examined. Enhanced surveillance for opioid overdose deaths facilitates the classification of deaths involving prescription and illicit opioids as well as identifying missed opportunities for prevention and response. Further information on CDC’s state efforts and opioid overdose data is available at https://www.cdc.gov/drugoverdose/index.html.

## References

[1] Seth P, Scholl L, Rudd RA, Bacon S. Overdose deaths involving opioids, cocaine, and psychostimulants—United States, 2015–2016. MMWR Morb Mortal Wkly Rep 2018;67:349–58. 10.15585/mmwr.mm6712a1 29596405 PMC5877356

[2] CDC. Understanding the epidemic. Atlanta, GA: US Department of Health and Human Services; CDC, 2017. https://www.cdc.gov/drugoverdose/epidemic/index.html

